# Safe endoscopic colorectal stenting using a biliary balloon catheter: Balloon anchoring method

**DOI:** 10.1055/a-2714-2506

**Published:** 2025-10-22

**Authors:** Kuniyo Gomi, Yuichi Takano, Toshiyuki Endo, Dai Matsubara, Erika Yoshida, Misako Tohata, Masatsugu Nagahama

**Affiliations:** 1 26858 Division of Gastroenterology, Department of Medicine, Showa Medical University Fujigaoka Hospital, Yokohama, Japan

Endoscopic colorectal stenting is a procedure in which a self-expandable metallic stent (SEMS) is implanted to treat malignant colorectal stenosis. The clinical success rate for bridge-to-surgery stenting is reported to be 94%, with 2% perforation and 1.2% migration rates 1 . Because prior endoscopy is rarely performed, information about the proximal bowel at the obstruction site is often lacking. Therefore, contrast-enhanced computed tomography is important for evaluating presence of perforation, length of stenosis, and bowel configuration to ensure safe implantation.

Conventionally, a contrast catheter is used to assess the stenosis, but in some cases, identifying the proximal end is difficult due to dilated bowel. We adapted the “balloon anchoring method,” originally developed for duodenal strictures 2 , for colorectal use. This method employs a biliary balloon catheter typically used for bile duct stone extraction. The balloon is inflated in the dilated bowel proximal to the stenosis and pulled back until it contacts the stenosis, allowing clear visualization of the stenotic length and optimal stent placement.

We used this method to treat a 72-year-old woman with ascending colon cancer and obstructive symptoms. After gently advancing the endoscope using water infusion, a biliary balloon catheter was introduced over a guidewire, inflated in the dilated bowel, and retracted into the stenosis. This allowed us to measure the stenosis and determine stent position with high accuracy ( Video 1 ). This balloon anchoring method improves safety and precision of colorectal stenting, especially in cases with unclear proximal bowel anatomy.

We performed endoscopic colorectal stenting using the balloon anchoring method in a patient with colonic obstruction caused by ascending colon cancer.Video 1
